# The impact of hypertension on chronic kidney disease and end-stage renal disease is greater in men than women: a systematic review and meta-analysis

**DOI:** 10.1186/s12882-020-02151-7

**Published:** 2020-11-25

**Authors:** Misghina Weldegiorgis, Mark Woodward

**Affiliations:** 1 The George Institute for Global Health, Department of Epidemiology and Biostatistics, School of Public Health, Imperial College London, London, UK; 2 The George Institute for Global Health, University of New South Wales Sydney, Sydney, Australia; 3 Department of Epidemiology, Johns Hopkins University, Baltimore, MD USA

## Abstract

**Background:**

Hypertension (HTN) is an established risk factor for chronic kidney disease (CKD) and end-stage renal disease (ESRD). Whether sex differences in the effect of HTN on CKD and ESRD incidence exist remains unclear. This systematic review and meta-analysis was conducted to evaluate the relative impact of HTN on CKD and ESRD risk in women compared with men.

**Methods:**

We systematically searched Embase and PubMed for cohort studies until 24 July 2020. Studies were selected if they reported a sex-specific association between systolic blood pressure (SBP) and CKD or ESRD. Random effects meta-analyses with inverse variance weighting were used to pool sex-specific relative risks (RRs) and the women-to-men ratio of RRs (the RRR) for incident CKD and ESRD.

**Results:**

Data from six cohorts, including 2,382,712 individuals and 6856 incident CKD events, and 833 ESRD events, were included in the meta-analysis. The RR for incident CKD or ESRD associated with HTN (SBP ≥140 mmHg) versus ideal BP (SBP < 120 mmHg) was 1.56 (95% CI, 1.39–1.75) in women and 2.06 (95% CI, 1.64–2.60) in men. The RR for incident CKD or ESRD was 23% lower in women than in men RRR 0.77 [95% CI, 0.63–0.95] with no significant heterogeneity between studies (*p*-value for Q test = 0.507, *I*^*2*^ = 17.7%).

**Conclusion:**

HTN confers about a fifth lower excess risk of incident CKD or ESRD in women than men. Sex differences in onset, duration, and severity of some risk factors, such as albuminuria, diabetes, cardiovascular disease, obesity, and socioeconomic status, may explain part of the excess risk in men. Another explanation could be that women might be under-diagnosed and less likely to initiate dialysis. Future studies are needed to demonstrate the mechanisms responsible for the observed sex difference.

**Supplementary Information:**

The online version contains supplementary material available at 10.1186/s12882-020-02151-7.

## Background

Chronic kidney disease (CKD) is one of the leading public health problems that affect millions of women and men worldwide [1, 2]. Hypertension (HTN) is a crucial risk factor for the development of CKD [3], progression to end-stage renal disease (ESRD) [4], cardiovascular disease (CVD) [5], and mortality [6]. Accordingly, several guidelines recommend early detection and treatment of HTN to delay the disease’s progression and reduce its complications in both sexes [7, 8]. However, the extent to which women and men with HTN are at a similar risk of developing CKD outcomes has not been extensively examined.

A recent study suggested that CKD’s prevalence is higher in women than men [9], while another study indicated that the lifetime risk of ESRD is higher in men than women [10]. A meta-analysis including more than 11,000 patients from 68 cohort studies of the sex-specific effect of CKD progression in patients with nondiabetic CKD [11] suggested that women tend to progress to ESRD at a slower rate than men, irrespective of the aetiology. In contrast, a patient-level meta-analysis of 11 randomized trials that used angiotensin-converting enzyme inhibitors, in 1860 patients with CKD, concluded that the rate of renal disease progression might be faster among women than in men [12]. The differences in these studies’ results may be attributed to using different definitions of outcomes, the different study designs, and variations in patient populations, especially given that no study was explicitly designed to examine sex differences.

Evidence for any clinically meaningful sex differences in relationships between HTN and CKD and ESRD could provide insight into mechanisms and optimal approaches for managing and treating raised blood pressure in both men and women. Therefore, we conducted this systematic review and meta-analysis to evaluate the sex-specific association between prevalent HTN and CKD and ESRD incidence.

## Methods

### Search strategy

PubMed and Embase systematic search was performed for cohort studies until 24 July 2020, using a combined text word and medical subject heading (MeSH) search strategy (Supplemental Methods S1). To identify other potentially relevant studies, references were scanned. Observational cohort studies were included in the meta-analysis if they had reported relative risks (RRs) or equivalents for CKD or ESRD for both men and women with HTN compared with ideal systolic blood pressure (SBP). Data were extracted from the adjusted model. Studies were excluded if they did not report such estimates or did not provide information about variability around the point estimate. The search strategy and items for the extraction of data were predefined and agreed upon by both authors (M. Weldegiorgis and M. Woodward). Both authors conducted the literature search independently. Doubts concerning the inclusion or exclusion of articles and data extraction were discussed by both authors and settled by mutual consent. This study was conducted in accordance with the Preferred Reporting Items for Systematic Reviews and Meta-Analyses (PRISMA) statement [13], Supplemental Methods S2. The included studies’ quality was assessed using the Newcastle-Ottawa Scale (Supplemental Methods S3 & S4) [14].

### Predictor and outcomes

We assessed the impact of HTN (SBP ≥140 mmHg) compared to the ideal BP (SBP < 120 mmHg). The outcomes considered were CKD, defined as an estimated glomerular function (eGFR) < 60 mL/min/1.73m^2^/year or proteinuria ≥1+ determined by dipstick; and ESRD, defined as the initiation of dialysis, renal transplantation, or death due to kidney disease.

### Data extraction and statistical analysis

For every included study, the sex-specific RRs with 95% confidence intervals (CIs) were extracted for individuals with HTN versus those with ideal BP taking the maximal adjustment. The logarithm of the RR (lnRR) was pooled across studies using random-effects restricted maximum likelihood (REML) meta-analysis with inverse variance weighting and then back-transformed to obtain the pooled RR separately for women and men. Similarly, we pooled the differences of the lnRR across studies, then back-transformed the data to obtain the pooled women-to-men ratio of RRs (RRRs) and the corresponding 95% CIs. The standard error (SE) of the RR for each sex was computed using (a) lnRR and the upper (lnUL) and lower limit (lnLL) of the CIs, (b) taking the mean of the SE of the lnLL and LnUL ((lnRR-lnLL)/1.96 + (lnUL−lnRR)/1.96)/2. The SE of the lnRRR was computed as the square root of the sum of the variance of the two sex-specific lnRRs [15]. To assess heterogeneity between cohorts, we computed *I*^*2*^ statistics and Cochran’s Q tests. *I*^*2*^ is classified in to three levels, < 30% (low), 30 to 60% (moderate), and > 60% (substantial). To measure inter-cohort variance, we calculated *τ*^*2*^. Furthermore, to account for *τ*^*2*^ in uncertainty around the pooled estimates, we computed 95% prediction intervals for the RRs [16]. ﻿ In the sensitivity analysis, we calculated the pooled RR only for the CKD outcomes and also for studies with a follow-up time of greater than 5 years. We used random-effects meta-regression analysis to evaluate whether differences in the duration of study follow-up contributed to heterogeneity between studies. Funnel plot was used to assess the presence of publication bias by plotting the natural log of the RRRs against its standard error. We used R version 3.2.2 (www.R-project.org) and Metafor package to analyze the data, and two-tailed *p*-values < 0.05 were considered statistically significant.

## Results

**Fig. 1:**
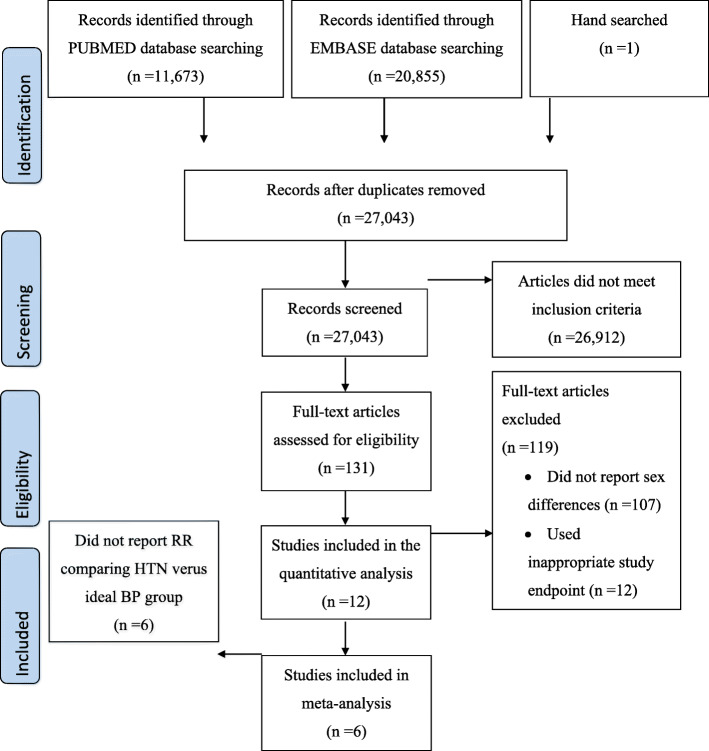
Flow chart of the study selection

**Fig. 2:**
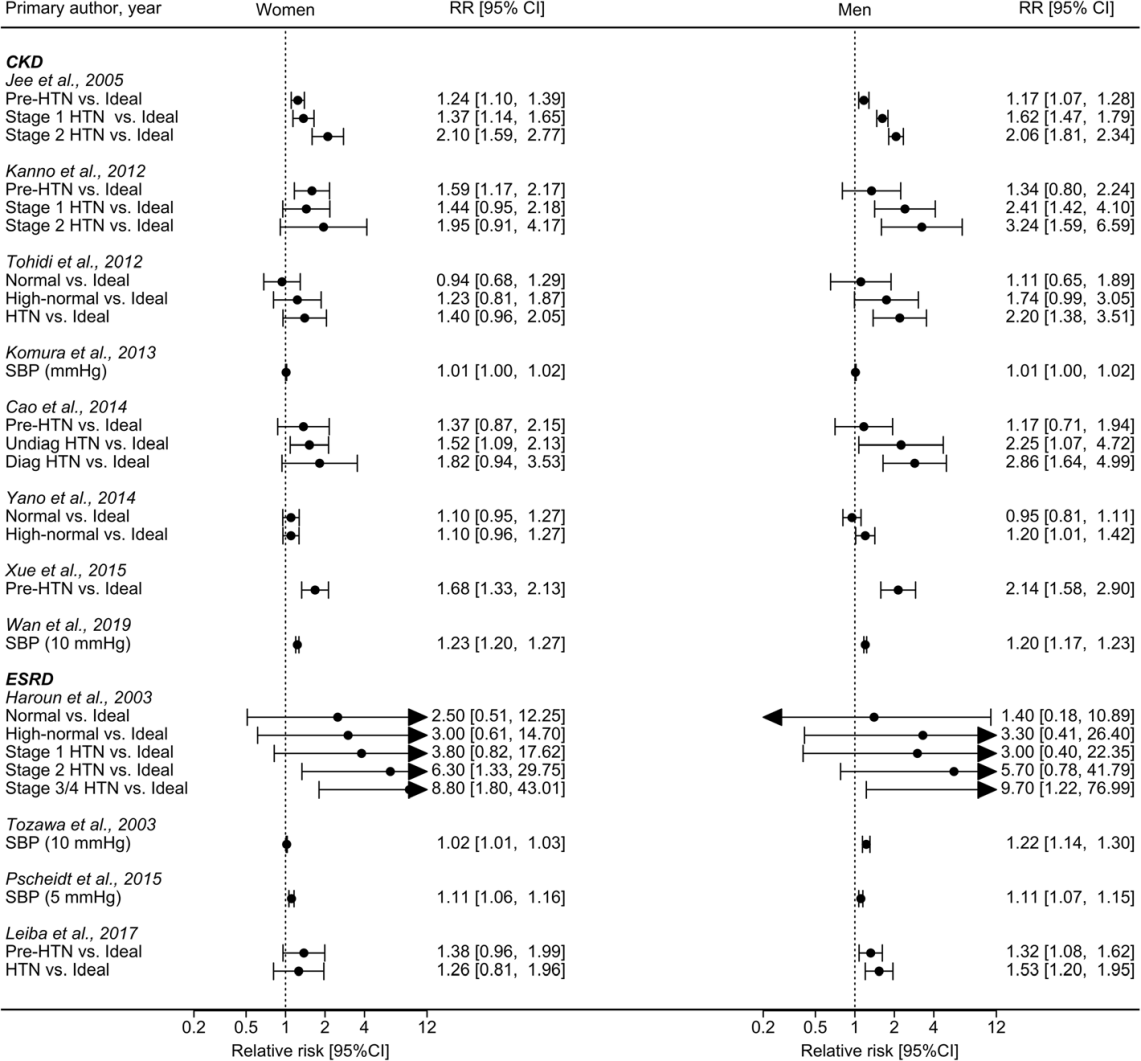
Summary of the maximum-adjusted relative risks and 95% confidence intervals, by study, showing all the blood pressure comparisons analyzed. We categorized the reported systolic blood pressure thresholds from each study as Ideal, SBP < 120 mmHg; Normal, SBP 120–129 mmHg; High-normal, SBP 130–139 mmHg; Prehypertension, SBP 120–139 mmHg; Hypertension, SBP ≥140 mmHg; Stage 1 Hypertension, SBP 140–159 mmHg; Stage 2 Hypertension, SBP 160–179 mmHg; Stage 3/4 Hypertension, SBP ≥180 mmHg

Of the 27,043 articles that were identified through the systematic search, 131 qualified for full-text evaluation (Fig. 1). In total, twelve papers reported on sex differences in the association between SBP and the risk of CKD or ESRD (Table 1). Figure 2 shows the summary of the maximum-adjusted RRs and 95% CIs, by study, showing all the BP comparisons analyzed. Of these, six studies (2,382,712 individuals and 6856 incident CKD events and 833 ESRD events) that compared HTN versus ideal BP were included in the meta-analysis. These studies were from Korea, Japan, Iran, China, the US, and Israel [17–19, 21, 25, 28]. The individuals were between 20 and 70 years of age at study baseline, and the duration of follow-up ranged from 4.5 to 20 years across studies. All studies were adjusted for age, and most were adjusted for body-mass index and smoking status (Supplemental Table S1).

**Table 1:** Characteristics of studies included in quantitative analysis

						Systolic blood pressure categories (mmHg)					
Primary author, year	Country	Baseline study, year	Follow-up, years	N (% Female)	Age, year	< 120n (% Female)	120–129 n (% Female)	130–139n (% Female)	≥140n (% Female)	Total events (% Female)	
**Jee et al (2005)**^a,^ [17]	Korea	1990–1992	10^b^	157,377 (33.6)	35–59^d^	55,940 (53.3)	67,307 (26.2)		34,135 (15.8)	5478 (27.9)	***CKD***
**Kanno et al (2012)**^a,^ [18]	Japan	1993–2007	6.5^b^	2150 (63.4)	60.3^b^	586 (75.8)	815 (61.2)		749 (56.2)	461 (62.7)	
**Tohidi et al (2012)**^a,^ [19]	Iran	1999–2001	9.9^b^	3313 (56.1)	≥ 20^b^	NA	NA	NA	NA	723 (71.5)	
Komura et al. (2013) [20]	Japan	1999	10^b^	1506 (68.6)	58.2^b^	NA	NA	NA	NA	466 (28.9)	
**Cao et al (2014)**^a,^ [21]	China	2006–2011	4.5^c^	1703 (46)	45.6^b^	828 (64.2)	546 (31.7)		329 (24.0)	194 (31)	
Yano et al. (2014) [22]	Japan	2008	3^b^	42,625 (63.8)	60^c^	17,759 (68.6)	14,064 (60.9)	10,802 (59.5)	NA	2142 (57.4)	
Xue et al. (2015) [23]	China	2006–2007	3.9^c^	32,385 (27.2)	46.4^b^	12,351 (36.3)	20,034 (21.6)		NA	601 (54.9)	
Wan et al. (2019) [24]	China	2011–2012	6^c^	156,469 (58.5)	64.3^b^	NA	NA		NA	﻿30,993 (NA)	
**Haroun et al (2003)**^a,^ [25]	US	1974	20^b^	23,534 (59.2)	NA	3686 (78.8)	5333 (65.7)	4513 (54.8)	10,002 (50.5)	143 (NA)	***ESRD***
Tozawa et al. (2003) [26]	Japan	1983–1984	17^b^	98,759 (52.5)	50^b^	23,187 (64.8)	22,835 (48.9)	16,840 (45.9)	35,897 (50.1)	400 (42.3)	
Pscheidt et al. (2015) [27]	Austria	1988–2005	17.5^b^	185,341 (53.9)	38.9^c^	117,658 (NA)	NA	NA	67,506 (NA)	403 (39.2)	
**Leiba et al (2017)**^a,^ [28]	Israel	1977–2013	16.8^c^	2,194,635 (41.7)	16–19^d^	1,465,733 (43.4)	442,077 (36.5)		286,825 (41)	690 (23.3)	

*Abbreviation: N* Total number of individuals in the study, *n* Number of individuals in each blood pressure category, *SD* Standard deviation, *IQR* Interquartile range, *NA* Not available. *Note:* ^a^ studies included in the meta-analysis (in bold); ^b^, mean; ^c^, median; ^d^, range

**Fig. 3:**
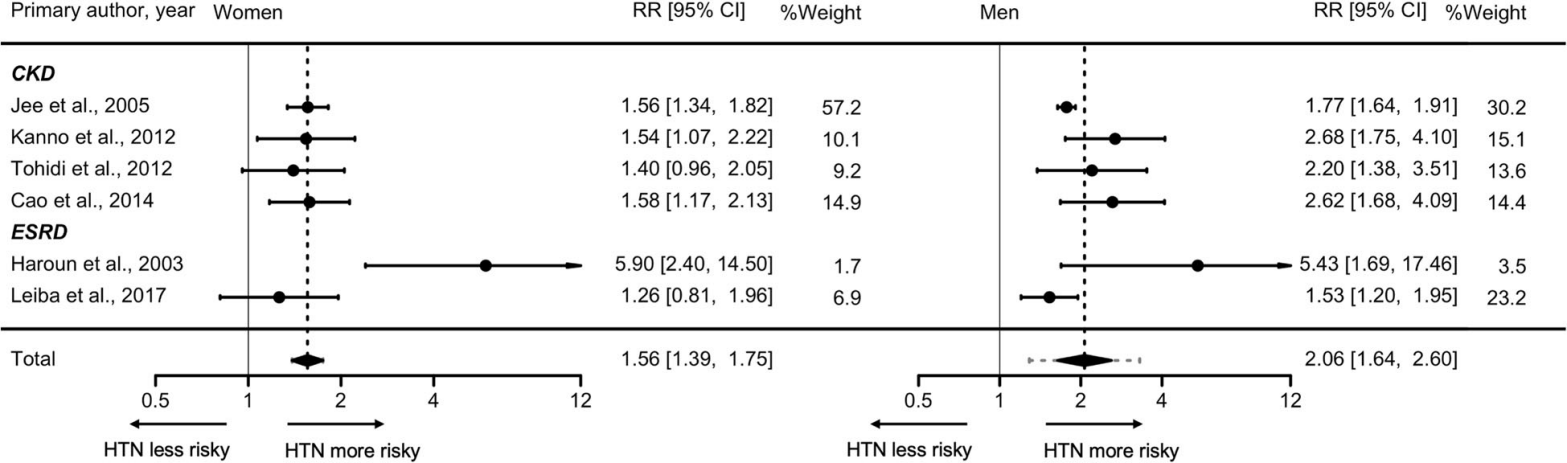
The maximum-adjusted pooled relative risk and 95% confidence intervals for chronic kidney disease and end-stage renal disease in women (left panel) and men (right panel), comparing individuals with Hypertension versus ideal blood pressure

**Fig. 4:**
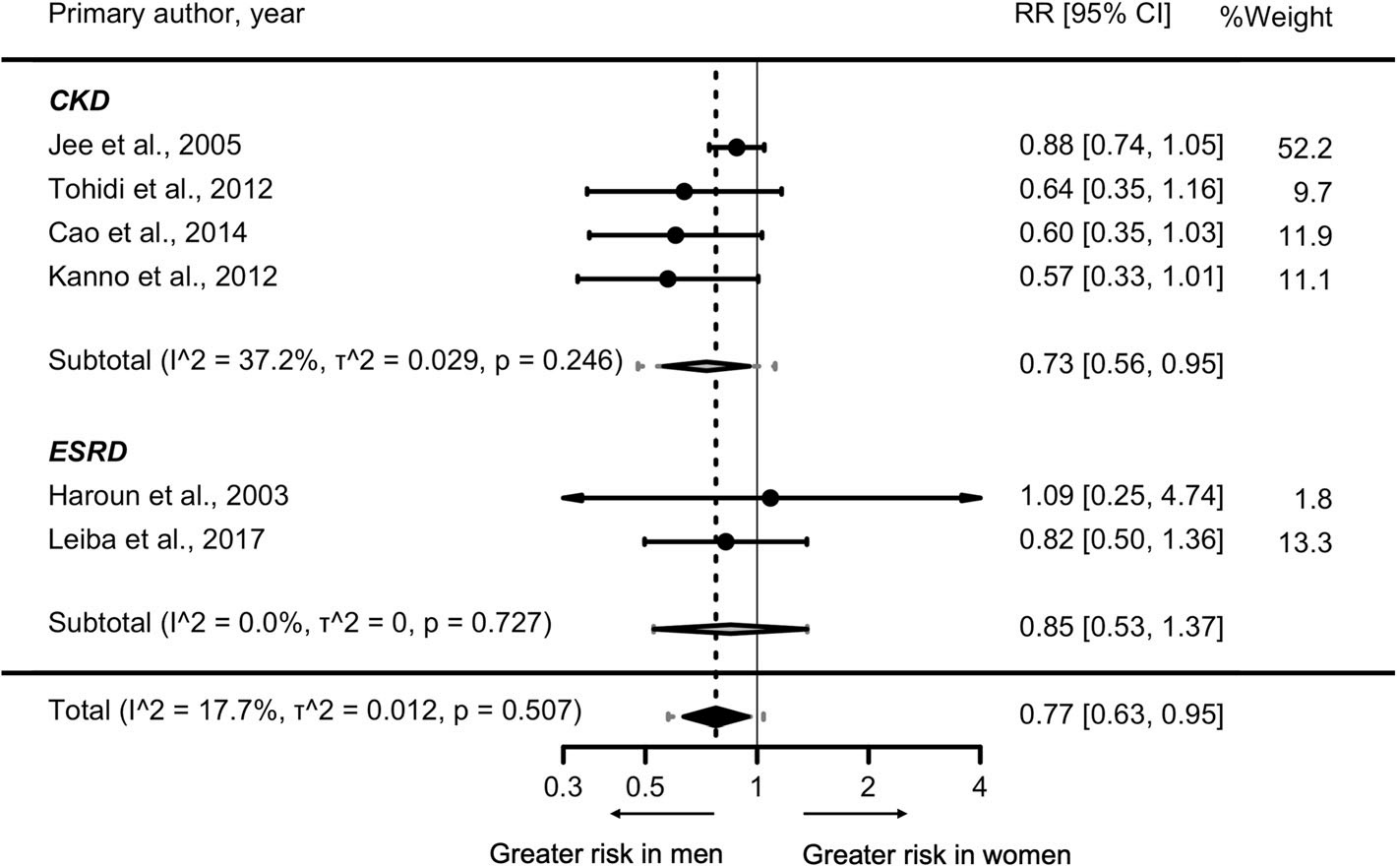
The maximum-adjusted women-to-men relative risk ratio and 95% confidence intervals for chronic kidney disease and end-stage renal disease, comparing individuals in Hypertension versus ideal blood pressure

The RR for incident CKD or ESRD associated with HTN versus ideal BP was 1.56 (95% CI, 1.39–1.75) in women (*I*^*2*^ = 0%, *﻿τ*^*2*^ = 0, *p*-value for Q test = 0.087) and 2.06 (95% CI, 1.64–2.60) in men (*I*^*2*^ = 66.5%, ﻿*τ*^*2*^ = 0.044, *p*-value for Q test = 0.033) (Fig. 3). The results were similar when we limited the analysis to only the CKD outcome (Supplemental Fig. S1) and studies with a follow-up time of greater than 5 years (Supplemental Fig. S2). The women to men RRR for incident CKD or ESRD was 0.77 [95% CI, 0.63–0.95] with no significant heterogeneity between studies (*I*^*2*^ = 17.7%, *τ*^*2*^ = 0.012, *p*-value for Q test = 0.507) (Fig. 4) as well as no evidence of publication bias (Egger’s test, *p*-value = 0.203; Supplemental Fig. S3). The pooled RRR did not vary significantly by the duration of study follow-up (*p*-value = 0.087; Supplemental Fig. S4).

## Discussion

In this pooled analysis of six cohorts, with data for more than two million individuals and 7689 CKD or ESRD events, HTN was a stronger risk factor for CKD and ESRD in men than women. Compared with men with HTN, women with HTN had a 23% lower relative risk for CKD or ESRD. This finding may have important implications for improving risk stratification and preventive strategies of CKD and ESRD in the general population.

Recent studies have focused on pre-HTN rather than HTN, and the evidence about sex difference in risk of CKD and ESRD is conflicting [3, 29, 30]. A meta-analysis that included six prospective cohort studies found a strong association between pre-HTN and increased long-term ESRD risk. In subgroup analysis, the study demonstrated that females with pre-HTN had a relatively higher risk of ESRD than their male counterparts; however, the difference was not statistically significant [29]. In contrast, another meta-analysis, including seven cohort studies, found a higher risk of CKD in prehypertensive men than women [31]. Further, a recent meta-analysis of 16 cohort studies found that HTN and pre-HTN to be independent predictors of decreased eGFR in the general population. However, the result was not stratified by sex [3].

Access to timely and good-quality health care is an important modifiable factor that may cause a considerable disparity in CKD’s risk profile between women and men [32]. Limited access to medical care may result in delayed CKD diagnosis, inadequate education in diet and self-care, insufficient access to medication or monitoring, and suboptimal treatment and follow-up. Interestingly, intensive blood pressure-lowering medications may also lead to a higher risk of acute kidney injury and CKD progression [33, 34]. Presently, our knowledge about sex differences in the diagnosis and management of hypertension is limited; not all patients may be receiving adequate guideline-recommended care. Therefore, a greater understanding of how sex contributes to the variability of CKD burden may have a substantial impact on the overall CKD related morbidity and mortality.

Consistent with our study, the latest report from the United States Renal Data System (USRDS) showed lower ESRD incidence for women in nearly all countries [31]. Similarly, another study observed considerably fewer women than men being treated with hemodialysis for ESRD in 12 of the countries participating in the Dialysis Outcomes and Practice Patterns Study (DOPPS) from 2002 to 2012. The study also found that the average eGFR at hemodialysis initiation was higher in men than women [35]. This finding is supported by a recent pooled analysis of the Evaluating Prevention of Progression In Chronic kidney disease (EPPIC) trials, where women tend to start dialysis at an average eGFR value of 9 mL/min/1.73m^2^/year while men started at an average eGFR value of more than 11 mL/min/1.73m^2^/year [36]. This difference in the time to initiation of dialysis could be partly related to women having less access to nephrology care [32], they are less aware of their disease and the degree of its severity [37] or be more likely to choose conservative treatment [38–41].

Another possibility is that men may have poor adherence to antihypertensive medications, poor lifestyle choices, and more preexisting conditions that could put them at high risk of ESRD and death. A Norwegian study that followed more than 3000 patients with CKD demonstrated higher mortality and ESRD risks for men than for women [42]. Likewise, an individual meta-analysis of about two million patients from 46 cohorts showed that the risk of mortality is higher among men for all levels of eGFR rate and albuminuria levels. However, among patients with lower values of eGFR and patients with higher albuminuria levels, the elevation in mortality risk is steeper for women [43].

Biologically, the effect of sex hormones in mediating hypertension and CKD remains uncertain, while some evidence suggests a protective effect of estrogen [44, 45], further evidence implicates testosterone as having a role in renal injury [46, 47]. However, there is also evidence that oral contraceptive use in premenopausal women, and estrogen replacement therapy in postmenopausal women, are both associated with an increased risk of microalbuminuria [48]. In our study, information on menopausal status and use of hormone replacement therapy was not available, so we are unable to evaluate whether they had any modifying effect on the association between HTN and the risk of CKD in women.

Our study has several strengths. A comprehensive literature search was conducted to include studies that assessed the sex-specific impact of HTN on CKD and ESRD. The studies included in the analysis had a long duration of follow-up and a large sample size, enabling a more accurate assessment of associations. The study was restricted to cohort studies with reported multivariate-adjusted relative risks. Five out of the six studies included in our meta-analysis excluded individuals with prevalent CKD [17–19, 21, 28], thus it is less likely the associations observed in our meta-analysis result from confounding by unmeasured pre-existing kidney disease. Our study also had some limitations. The review protocol was not registered with the ﻿international prospective register of systematic reviews (http://www.crd.york.ac.uk/PROSPERO). We used only two databases (PubMed and Embase) for our systematic search. Due to the scarcity of sex-specific cohort studies, a limited number of studies were available for analysis. We had no access to individual patient-level data. Data on mortality were not available; therefore, there is a possibility of the potential effect of competing risk and survival bias. Further limitations include a lack of standardization in the level of adjustment for confounding and the intensity of CKD risk factor management across studies. However, analyses for women and men used the same adjustments, since we selected only studies with data on both sexes.

## Conclusion

This study demonstrated unequal impact of HTN on CKD and ESRD in men versus women, to the disadvantage of men. This disparity is unlikely to be explained by biological differences alone.

## Supplementary Information

**Additional file 1: Supplemental Methods S1.** Search strategies

**Additional file 2:** **Supplemental Methods S2.** PRISMA Checklist

**Additional file 3:** **Supplemental Methods S3.** Quality criteria according to a modified version of the Newcastle-Ottawa Quality assessment scale (*studies received one point for the achievement of these criteria)

**Additional file 4:** **Supplemental Methods S4.** Quality assessment of the studies included in the meta-analyses

**Additional file 5:** **Supplemental Table S1**. Description of the characteristics of the studies included in the quantitative analysis

**Additional file 6:** **Supplemental Figure S1**. The maximum-adjusted pooled relative risk and 95% confidence intervals for chronic kidney disease in women (left panel) and men (right panel), comparing individuals with Hypertension versus ideal blood pressure

**Additional file 7:** **Supplemental Figure S2**. The maximum-adjusted pooled relative risk and 95% confidence intervals for chronic kidney disease and end-stage renal disease in women (left panel) and men (right panel), comparing individuals with Hypertension versus ideal blood pressure in studies with a follow-up time of greater than 5 year

**Additional file 8:** **Supplemental Figure S3**. Funnel plot with pseudo 95% confidence limits for the data in Fig. 4

**Additional file 9:** **Supplemental Figure S4**. Meta-regression of the maximum-adjusted women-to-men relative risk ratio versus duration of follow-up. For each study, the circles are drawn in proportion to the inverse variance

## Data Availability

All data generated or analyzed during this study are included in this published article. A list of commands for statistical analysis and data used for the meta-analysis are available on Open Science Framework (osf.io).

## Abbreviations

HTN: Hypertension
CKD: Chronic Kidney Disease;
ESRD: End-stage Renal Disease
BP: Blood Pressure
SBP: Systolic Blood Pressure
RR: Relative Risk
RRR: Relative Risk Ratio
CI: Confidence Interval
SE: Standard Error
lnRR: Logarithm of the Relative Risk
lnUL: Logarithm of the Upper Limit
lnLL: Logarithm of the Lower Limit
lnRRR: Logarithm of the Relative Risk Ratio
CVD: Cardiovascular Disease
MeSH: Medical Subject Heading
PRISMA: Preferred Reporting Items for Systematic Reviews and Meta-Analyses
eGFR: Estimated Glomerular Filtration Rate
REML: Restricted Maximum Likelihood
USRDS: United States Renal Data System
DOPPS: Dialysis Outcomes and Practice Patterns Study
EPPIC: Evaluating Prevention of Progression In Chronic kidney disease
PROSPERO: International Prospective Register of Systematic Reviews
