# Correction to: The impact of hypertension on chronic kidney disease and end-stage renal disease is greater in men than women: a systematic review and meta-analysis

**DOI:** 10.1186/s12882-020-02199-5

**Published:** 2020-12-21

**Authors:** Misghina Weldegiorgis, Mark Woodward

**Affiliations:** 1 The George Institute for Global Health, Department of Epidemiology and Biostatistics, School of Public Health, Imperial College London, London, UK; 2 The George Institute for Global Health, University of New South Wales Sydney, Sydney, Australia; 3 Department of Epidemiology, Johns Hopkins University, Baltimore, MD USA

**Correction to: BMC Nephrol (2020) 21:506**

**https://doi.org/10.1186/s12882-020-02151-7**

Following publication of the original article [1], the authors informed us that there are some errors due to a typesetting mistake. The corrections are as follows:

1. Figure 1 has been updated as the arrows in the figure were distorted during the production process.
2. The Supplemental Table S1 and Supplemental Methods S4 have been updated in accordance with the journal style.
3. The Supplementary Figure 1 and Supplementary Figure 2 have been updated with a better quality.

The original article has been corrected.
