# Supplementary material for: The impact of hypertension on chronic kidney disease and end-stage renal disease is greater in men than women: a systematic review and meta-analysis
Source: BMC Nephrol. 2020 Nov 25;21:506. doi: 10.1186/s12882-020-02151-7 (PMC7687699; doi:10.1186/s12882-020-02151-7)
Supplement: Supplementary file 1 — Additional file 1: Supplemental Methods S1. Search strategies [file 12882_2020_2151_MOESM1_ESM.docx]

**Supplemental Methods S1.** Search strategies

Report of search strategies as conducted in July 2020.

1. PubMed

Search period: unlimited

Date of search: 24.07.2020

| Step | Hits | Search |
| --- | --- | --- |
| #1 | 470,023 | "blood pressure" [Mesh] OR "arterial pressure" [Mesh] OR "hypertension" [Mesh] OR "systolic blood pressure" [MeSH] |
| #2 | 628,560 | blood pressure [tiab] OR arterial pressure [tiab] OR hypertension [tiab] OR systolic blood pressure [tiab] OR systolic BP [tiab] |
| #3 | 784,862 | #1 OR #2 |
| #4 | 815,765 | "sex" [MeSH] OR sex [tiab] OR gender [tiab] |
| #5 | 5,928,281 | ("men" [MeSH] OR men [tiab] OR "male" [MeSH] OR male [tiab]) AND ("women" [MeSH] OR women [tiab] OR "female" [MeSH] OR female [tiab]) |
| #6 | 6,144,423 | #4 OR #5 |
| #7 | 264,167 | "renal insufficiency, chronic" [Mesh] OR "kidney failure, chronic" [Mesh] OR "renal replacement therapy" [MeSH] |
| #8 | 285,788 | chronic kidney disease [tiab] OR renal failure [tiab] OR end stage kidney disease [tiab] OR dialysis [tiab] OR kidney transplant* [tiab] OR renal transplant* [tiab] |
| #9 | 45,733 | CKD [tiab] OR ESKD [tiab] OR ESKF [tiab] OR ESRF [tiab] OR ESRD [tiab] |
| #10 | 386,943 | #7 OR #8 OR #9 |
| #11 | 3,665,849 | "Cohort Studies" [Mesh] OR "Prospective Studies" [Mesh] OR "Longitudinal Studies"[Mesh] OR "follow-up studies" [Mesh] OR "Health Records, Personal" [Mesh] OR "Electronic Health Records" [Mesh] OR cohort [tiab] OR prospective [tiab] OR longitudinal Studies [tiab] OR follow-up [tiab] OR follow up [tiab] OR followed [tiab] OR health record* [tiab] OR medical record* [tiab] OR computerized record* [tiab] |
| #12 | 11,673 | #3 AND #6 AND #10 AND #11 |

2. Embase

Search period: unlimited

Date of search: 24.07.2020

| Step | Hits | Search |
| --- | --- | --- |
| #1 | 1,102,832 | exp blood pressure/ OR exp arterial pressure/ OR exp hypertension/ OR exp systolic blood pressure/ |
| #2 | 887,415 | blood pressure.tw. OR arterial pressure.tw. OR hypertension.tw. OR systolic blood pressure.tw. OR systolic BP.tw. |
| #3 | 1,319,456 | #1 OR #2 |
| #4 | 1,209,314 | sex/ OR gender/ OR sex.tw. OR gender.tw. |
| #5 | 6,375,822 | (exp men/ OR men.tw. OR exp male/ OR male.tw.) AND (exp women/ OR women.tw. OR exp female/ OR female.tw.) |
| #6 | 6,751,914 | #4 OR #5 |
| #7 | 270,286 | exp chronic kidney disease/ OR exp chronic kidney failure/ OR exp renal replacement therapy/ OR exp end stage renal disease/ |
| #8 | 403,288 | chronic kidney disease.tw. OR renal failure.tw. OR end stage kidney disease.tw. OR dialysis.tw. OR kidney transplant*.tw. OR renal transplant*.tw. |
| #9 | 76,532 | CKD.tw. OR ESKD.tw. OR ESKF.tw. OR ESRF.tw. OR ESRD.tw. |
| #10 | 524501 | #7 OR #8 OR #9 |
| #11 | 4,635,917 | exp cohort studies/ OR exp prospective studies/ OR exp longitudinal studies/ OR exp follow up/ OR exp electronic patient record/ OR exp electronic health record/ OR cohort.tw. OR prospective.tw. OR longitudinal Studies.tw. OR follow-up.tw. OR follow up.tw. OR followed.tw. OR health record*.tw. OR medical record*.tw. OR computerized record*.tw. |
| #12 | 20,855 | #3 AND #6 AND #10 AND #11 |

3. Combined search strategy

Search ((((((("blood pressure"[Mesh] OR "arterial pressure"[Mesh] OR "hypertension"[Mesh] OR "systolic blood pressure"[MeSH]))) OR ((blood pressure[tiab] OR arterial pressure[tiab] OR hypertension[tiab] OR systolic blood pressure[tiab] OR systolic BP[tiab])))) AND (((("sex"[MeSH] OR sex[tiab] OR gender[tiab]))) OR ((("men"[MeSH] OR men[tiab] OR "male"[MeSH] OR male[tiab]) AND ("women"[MeSH] OR women[tiab] OR "female"[MeSH] OR female[tiab]))))) AND ((((("renal insufficiency, chronic"[Mesh] OR "kidney failure, chronic"[Mesh] OR "renal replacement therapy"[MeSH]))) OR ((chronic kidney disease[tiab] OR renal failure[tiab] OR end stage kidney disease[tiab] OR dialysis[tiab] OR kidney transplant*[tiab] OR renal transplant*[tiab]))) OR ((CKD[tiab] OR ESKD[tiab] OR ESKF[tiab] OR ESRF[tiab] OR ESRD[tiab])))) AND (("Cohort Studies"[Mesh] OR "Prospective Studies"[Mesh] OR "Longitudinal Studies"[Mesh] OR "follow-up studies"[Mesh] OR "Health Records, Personal"[Mesh] OR "Electronic Health Records"[Mesh] OR cohort[tiab] OR prospective[tiab] OR longitudinal Studies[tiab] OR follow-up[tiab] OR follow up[tiab] OR followed[tiab] OR health record*[tiab] OR medical record*[tiab] OR computerized record*[tiab]))
