# Supplementary material for: The impact of hypertension on chronic kidney disease and end-stage renal disease is greater in men than women: a systematic review and meta-analysis
Source: BMC Nephrol. 2020 Nov 25;21:506. doi: 10.1186/s12882-020-02151-7 (PMC7687699; doi:10.1186/s12882-020-02151-7)
Supplement: Supplementary file 3 — Additional file 3: Supplemental Methods S3. Quality criteria according to a modified version of the Newcastle-Ottawa Quality assessment scale (*studies received one point for the achievement of these criteria) [file 12882_2020_2151_MOESM3_ESM.docx]

**Supplemental Methods S3:** Quality criteria according to a modified version of the Newcastle-Ottawa Quality assessment scale (*studies received one point for the achievement of these criteria):

Selection

S1) Representativeness of the exposed cohort

a) truly representative of the general population*****

b) somewhat representative of the general population

c) selected group e.g. patient groups

d) no description of the derivation of the cohort

S2) Selection of the non exposed cohort

a) drawn from the same community as the exposed cohort *****

b) drawn from a different source

c) no description of the derivation of the non exposed cohort

S3) Ascertainment of exposure

a) secure record (validated events) *****

b) structured interview

c) written self report

d) no description

S4) Demonstration that outcome of interest was not present at start of study

a) yes *****

b) no

Comparability

C1) Comparability of cohorts on the basis of the design or analysis

a) study controls for age*****

b) study does control for additional factors*****

Outcome

O1) Assessment of outcome

a) independent blind assessment or record linkage of fatal and non-fatal events*****

b) independent blind assessment or record linkage of fatal events

c) self report

d) no description

O2) Was follow-up long enough for outcomes to occur

a) yes (at least 3 years) *****

b) no

O3) Adequacy of follow up of cohorts

a) complete follow up - all subjects accounted for *****

b) subjects lost to follow up unlikely to introduce bias - > 10% follow up, or description provided of those lost *****

c) follow up rate < 90% and no description of those lost

d) no statement
