# Supplementary material for: The impact of hypertension on chronic kidney disease and end-stage renal disease is greater in men than women: a systematic review and meta-analysis
Source: BMC Nephrol. 2020 Nov 25;21:506. doi: 10.1186/s12882-020-02151-7 (PMC7687699; doi:10.1186/s12882-020-02151-7)
Supplement: Supplementary file 4 — Additional file 4: Supplemental Methods S4. Quality assessment of the studies included in the meta-analyses [file 12882_2020_2151_MOESM4_ESM.docx]

**Supplemental Methods S4:** Quality assessment of the studies included in the meta-analyses

| **Study name** | **Scores** | | | | | | | | | |
| --- | --- | --- | --- | --- | --- | --- | --- | --- | --- | --- |
|  | **S1** | **S2** | **S3** | **S4** | **C1** | **O1** | **O2** | **O3** |  | **SUM** |
| Jee et al (2005)^‡20^ | 0 | 1 | 0 | 1 | 2 | 1 | 1 | 1 |  | **7** |
| Kanno et al (2012)^‡22^ | 1 | 1 | 1 | 1 | 2 | 1 | 1 | 1 |  | **9** |
| Tohidi et al (2012)^‡19^ | 1 | 1 | 1 | 1 | 2 | 1 | 1 | 1 |  | **9** |
| Cao et al (2014)^‡18^ | 0 | 1 | 1 | 1 | 2 | 1 | 1 | 1 |  | **8** |
| Haroun et al (2003)^‡21^ | 0 | 1 | 0 | 0 | 2 | 1 | 1 | 1 |  | **6** |
| Leiba et al (2017)^‡17^ | 1 | 1 | 1 | 1 | 2 | 1 | 1 | 1 |  | **9** |
