# Supplementary material for: The impact of hypertension on chronic kidney disease and end-stage renal disease is greater in men than women: a systematic review and meta-analysis
Source: BMC Nephrol. 2020 Nov 25;21:506. doi: 10.1186/s12882-020-02151-7 (PMC7687699; doi:10.1186/s12882-020-02151-7)
Supplement: Supplementary file 5 — Additional file 5: Supplemental Table S1. Description of the characteristics of the studies included in the quantitative analysis [file 12882_2020_2151_MOESM5_ESM.docx]

**Supplemental Table S1**. Description of the characteristics of the studies included in the quantitative analysis

| **Source** | **Diagnostic criteria**  **for BP category** | **No of BP categories** | **Maximum adjustment variables** | **Excluded baseline CKD** | **Definition of outcome** |
| --- | --- | --- | --- | --- | --- |
| ***Outcome: CKD*** |  |  |  |  |  |
| **Jee et al (2005)^‡^**^20^ | JNC 7 | 4 | Age, diabetes, BMI, cholesterol, and smoking | Yes | Proteinuria ≥1+ determined by dipstick |
| **Kanno et al (2012)^‡^** ^22^ | JNC 7 | 4 | Age, smoking, alcohol, obesity, CVD, diabetes mellitus, eGFR, hypercholesterolemia, antihypertensive treatment, number of follow-up examinations, and year of the baseline examination | Yes | eGFR <60 mL/min/1.73m^2^/year or  proteinuria ≥1+ (30 mg/dL) determined by dipstick |
| **Tohidi et al (2012)^‡^**^19^ | EGMAH 2007 | 4 | Age, eGFR, diabetes, marital status, CVD, education level, dyslipidemia, abdominal obesity, BMI, smoking, FHDM | Yes | eGFR <60 mL/min/1.73m^2^/year for >3 months |
| Komura et al (2013)^43^ | NA | NA | Female: Age, BMI, DBP, AST, TG, and creatinine. Male: Age, BMI, DBP, fasting blood glucose, AST, y-GTP, and creatinine. | Yes | eGFR <60 mL/min/1.73m^2^/year or  proteinuria ≥1+ (30 mg/dL) determined by dipstick |
| **Cao et al (2014)^‡^**^18^ | JNC 7 | 4 | Age, smoking, alcohol, obesity, diabetes mellitus, TC, and eGFR | Yes | eGFR <60 mL/min/1.73m^2^/year or  proteinuria ≥1+ (30 mg/dL) determined by dipstick |
| Yano et al (2014)^44^ | JNC 7 | 3 | Age and BMI | Yes | eGFR <60 mL/min/1.73m^2^/year or  proteinuria ≥1+ determined by dipstick |
| Xue et al (2015)^45^ | JNC 7 | 2 | Age, TG, LDL-C, HDL-C, FBG, and smoking, drinking, and diabetes | Yes | eGFR <60 mL/min/1.73m^2^/year |
| Wan et al (2019)^46^ | ICPC 2 K86/K87 | 7 | ﻿ Age, smoking, BMI, DBP, LDL-C, eGFR, the usages of ACE -inhibitor/ARB, β-blocker, CCB, diuretic, other antihypertensive drugs, lipid-lowering agent, and Charlson index at baseline, and adjusted with regression dilatation ratio | Yes | eGFR <60 mL/min/1.73m^2^/year |
| ***Outcome: ESRD*** |  |  |  |  |  |
| **Haroun et al (2003)^‡^**^21^ | JNC 6 | 6 | Age, smoking, and treated diabetes | No | Initiation of dialysis or transplantation, kidney disease on the death certificate & confirmed as CKD by medical record review |
| Tozawa et al (2003)^47^ | JSH 2000 | 1 | Age, BMI, and proteinuria | No | Initiation of chronic dialysis |
| Pscheidt et al (2015)^48^ | JNC 7 | 4 | Age, BMI, smoking, DBP, blood glucose, TG, TC, and gamma-GT | Yes | Initiation of dialysis or renal transplantation |
| **Leiba et al (2017)^‡^**^17^ | NHBPEP 4 | 3 | Age, birth year, BMI, origin, education, and socioeconomic status | Yes | Initiation of dialysis or renal transplantation |
| *Abbreviation:* EGMAH, European Guideline for the Management of Arterial Hypertension; JNC, The Joint National Committee on Prevention, Detection, Evaluation, and Treatment of High Blood Pressure; ICPC, International Classification of Primary Care; JSH, Japanese Society of Hypertension; NHBPEP, The National High Blood Pressure Education Program; BMI, body-mass index; CVD, cardiovascular disease; eGFR, estimated glomerular filtration rate; FHDM, family history of diabetes mellitus; DBP, diastolic blood pressure; TG, triglyceride; LDL-C, low-density lipoprotein cholesterol; HDL-C, high-density lipoprotein cholesterol; FBG, fasting blood glucose; AST, aspartate aminotransferase; ALT, alanine aminotransferase; GTP, glutamyl transpeptidase; TC, total cholesterol; ACE, angiotensin-converting enzyme; ARB, angiotensin receptor blocker; CCB, calcium channel blocker. *Note:* **^‡^**, studies included in meta-analysis (in bold). | | | | | |
