# Supplementary figures and images for: The impact of hypertension on chronic kidney disease and end-stage renal disease is greater in men than women: a systematic review and meta-analysis
Source: BMC Nephrol. 2020 Nov 25;21:506. doi: 10.1186/s12882-020-02151-7 (PMC7687699; doi:10.1186/s12882-020-02151-7)

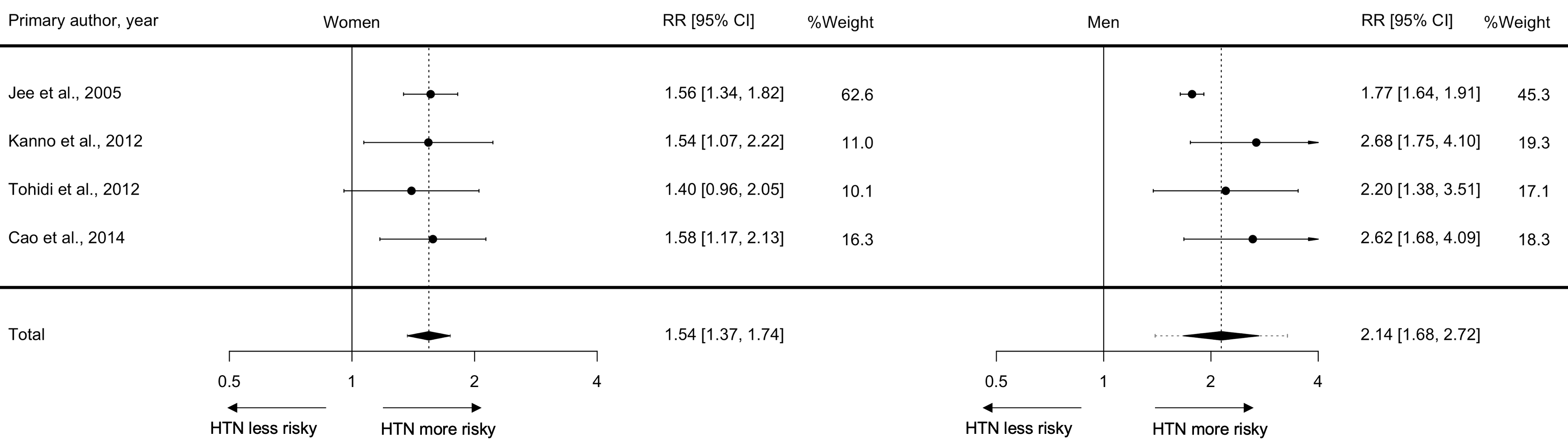

Supplement: Supplementary file 6 — Additional file 6: Supplemental Figure S1. The maximum-adjusted pooled relative risk and 95% confidence intervals for chronic kidney disease in women (left panel) and men (right panel), comparing individuals with Hypertension versus ideal blood pressure [file 12882_2020_2151_MOESM6_ESM.tiff]

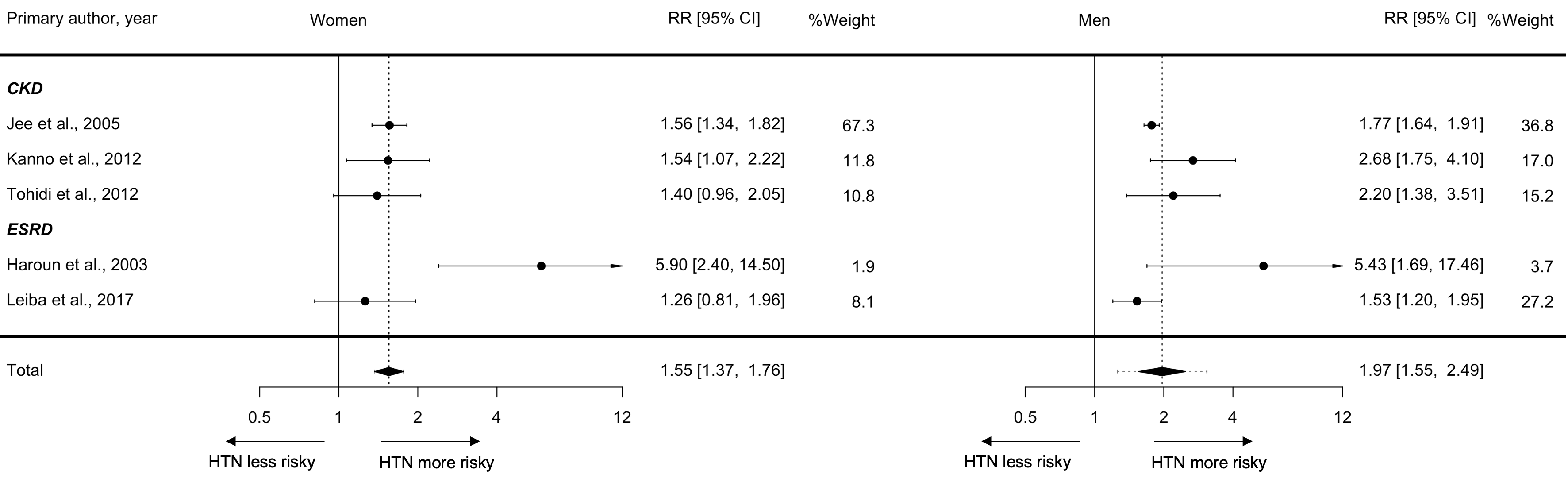

Supplement: Supplementary file 7 — Additional file 7: Supplemental Figure S2. The maximum-adjusted pooled relative risk and 95% confidence intervals for chronic kidney disease and end-stage renal disease in women (left panel) and men (right panel), comparing individuals with Hypertension versus ideal blood pressure in studies with a follow-up time of greater than 5 year [file 12882_2020_2151_MOESM7_ESM.tiff]

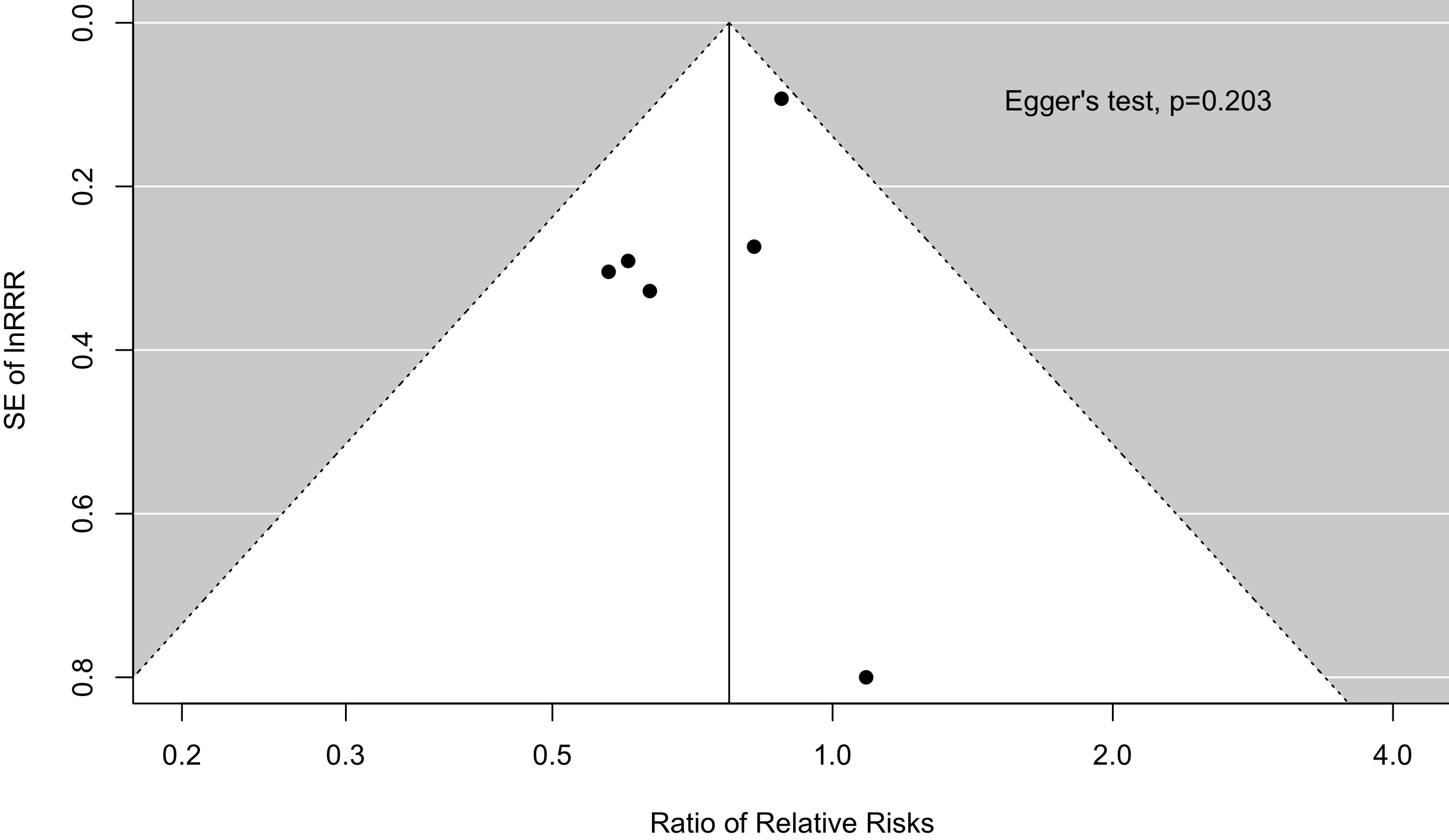

Supplement: Supplementary file 8 — Additional file 8: Supplemental Figure S3. Funnel plot with pseudo 95% confidence limits for the data in Fig. 4 [file 12882_2020_2151_MOESM8_ESM.tiff]

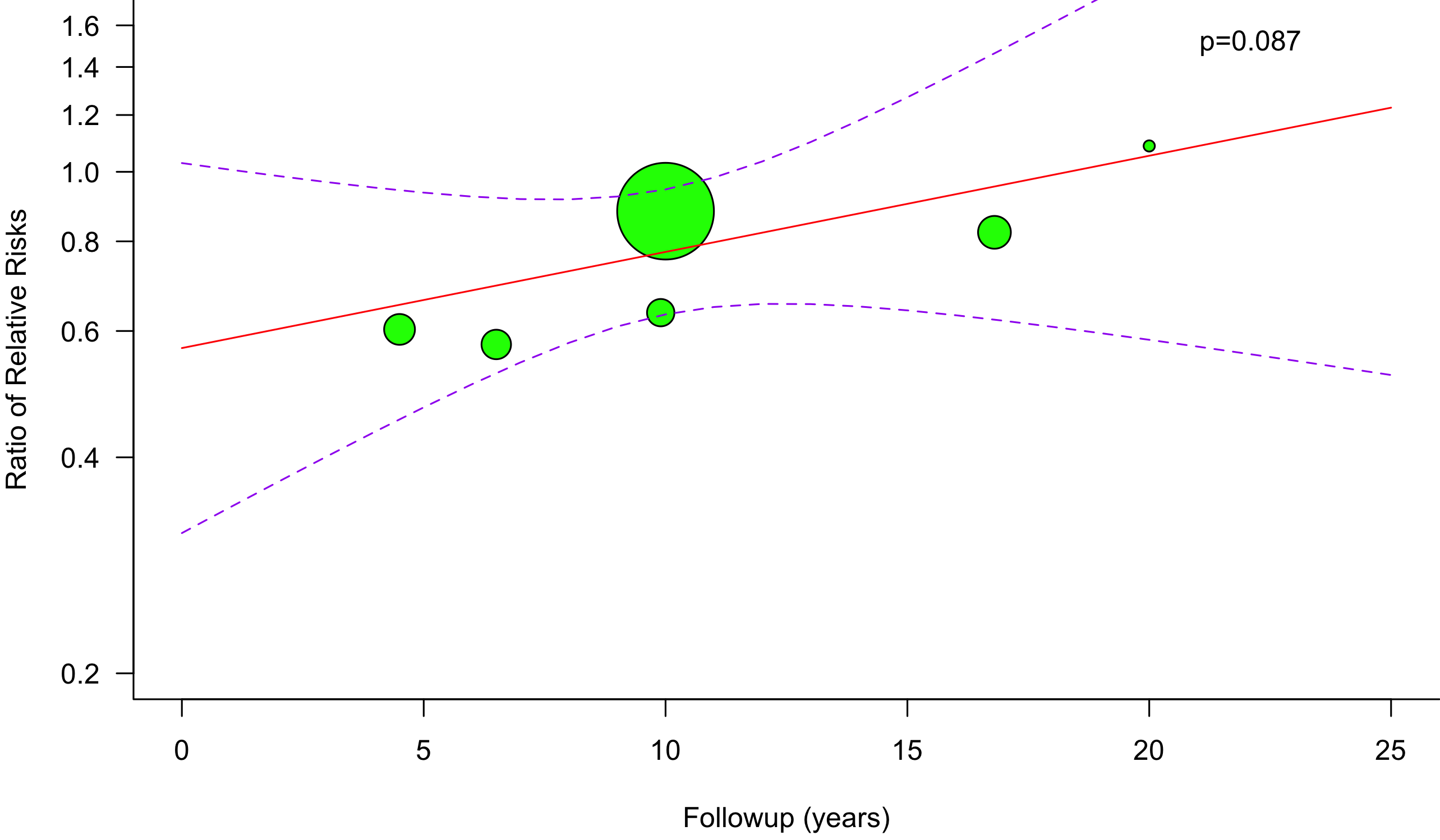

Supplement: Supplementary file 9 — Additional file 9: Supplemental Figure S4. Meta-regression of the maximum-adjusted women-to-men relative risk ratio versus duration of follow-up. For each study, the circles are drawn in proportion to the inverse variance [file 12882_2020_2151_MOESM9_ESM.tiff]
